# Dysregulation of developmental and cell type-specific expression of glycoconjugates on hematopoietic cells: a new characteristic of myelodysplastic neoplasms (MDS)

**DOI:** 10.1038/s41375-022-01784-x

**Published:** 2023-02-09

**Authors:** Margot F. van Spronsen, Sophie Horrevorts, Claudia Cali, Theresia M. Westers, Sofie Van Gassen, Yvan Saeys, Sandra J. van Vliet, Yvette van Kooyk, Arjan A. van de Loosdrecht

**Affiliations:** 1 Amsterdam UMC location Vrije Universiteit Amsterdam, Departement of Hematology, Cancer Center Amsterdam, Boelelaan, 1117 Amsterdam, The Netherlands; 2 Amsterdam UMC location Vrije Universiteit Amsterdam, Department of Molecular Cell Biology and Immunology, Cancer Center Amsterdam, Amsterdam Infection and Immunity Institute, Boelelaan, 1117 Amsterdam, The Netherlands; 3 VIB Inflammation Research Center, Ghent University, Ghent, Belgium; 4 Department of Applied Mathematics, Computer Science and Statistics, Ghent University, Ghent, Belgium

**Keywords:** Myelodysplastic syndrome, Acute myeloid leukaemia, Haematopoiesis

## To the Editor

Myelodysplastic neoplasms (MDS) are age-associated hematopoietic neoplasms characterized by myeloid dysplasia and cytopenias. Patients with MDS have a diverse clinical course, ranging from indolent conditions to acute myeloid leukemia (AML) [1]. Sequencing of leukocytes from MDS patients revealed somatic mutations that correlated with their clinical outcome. Studies that traced driver mutations back to hematopoietic stem and progenitor cells (HSPCs) supported the concept that myelodysplastic phenotypes arise from cancer stem cells [2]. Importantly, HSPC function and interaction with the bone marrow (BM) microenvironment depends partly on glycan-protein interactions [3]. Glycosylation is the post-translational modification by which oligosaccharide chains are covalently attached to amino acids or lipids. Glycoproteins are decorated with glycans at nitrogen- and oxygen atoms in the endoplasmic reticulum or Golgi apparatus, yielding *N*- and *O*-linked glycosylation, respectively. Aberrant glycosylation is a hallmark of oncogenesis and results in modulated inflammatory responses, apoptosis and cancer cell metastasis [4]. Insights into aberrant glycome structures have been applied for the development of biomarkers and therapeutic antibodies. Although described in other hematological malignancies, glycosylation is understudied in MDS [5, 6]. This study explored glycosignatures in MDS and AML to elucidate pathological mechanisms that could serve as biomarker.

This study was conducted following the Helsinki Declaration and approved by the Medical Ethics Committee of the Amsterdam UMC location Vrije Universiteit Amsterdam (VUmc 2014-100, VUmc 2019-3448). Samples were obtained from patients with MDS (*n* = 14, Table S1), AML (*n* = 9) and iron deficiency and dysregulated iron metabolism (IDef, *n* = 17) (Supplementary Information). Normal bone marrow (NBM, *n* = 10) was acquired from cardiothoracic surgery patients after written informed consent. We used plant lectins as probes to recognize glycoconjugates based on their specific glycan-binding affinities. Cells were stained with an antibody backbone and one of the following lectins: Phytohemagglutinin-L (PHA-L), Concanavalin A (ConA), *Maackia amurensis* agglutinin II (MAA-II), *Maackia amurensis* leucoagglutinin I (MAL-I) and *Sambucus nigra* agglutinin (SNA, Table S2). The lectins PHA-L, ConA and SNA recognize tetra-antennary *N*-glycans, high-mannose glycans and di-antennary *N*-glycans, and α2-6 sialoglycans, respectively. The MAA-II and MAL-I lectins bind to α2-3 sialoglycans with distinct carbohydrate binding specificities: MAA-II has a preference for *O*-linked α2-3 sialic acids and MAL-I for *N*-linked α2-3 sialic acids. Lectins were selected based on their binding to hematopoietic cells as demonstrated in a pilot study (data not shown). Flow cytometry data were manually pre-gated on CD45^+^ leukocytes, aggregated into a dataset of 40 ∙ 10^6^ cells and subjected to unsupervised clustering (Supplementary Information, Fig. S1). Statistics are described in the (Supplementary Information Tables S3–S5).

**Fig. 1:**
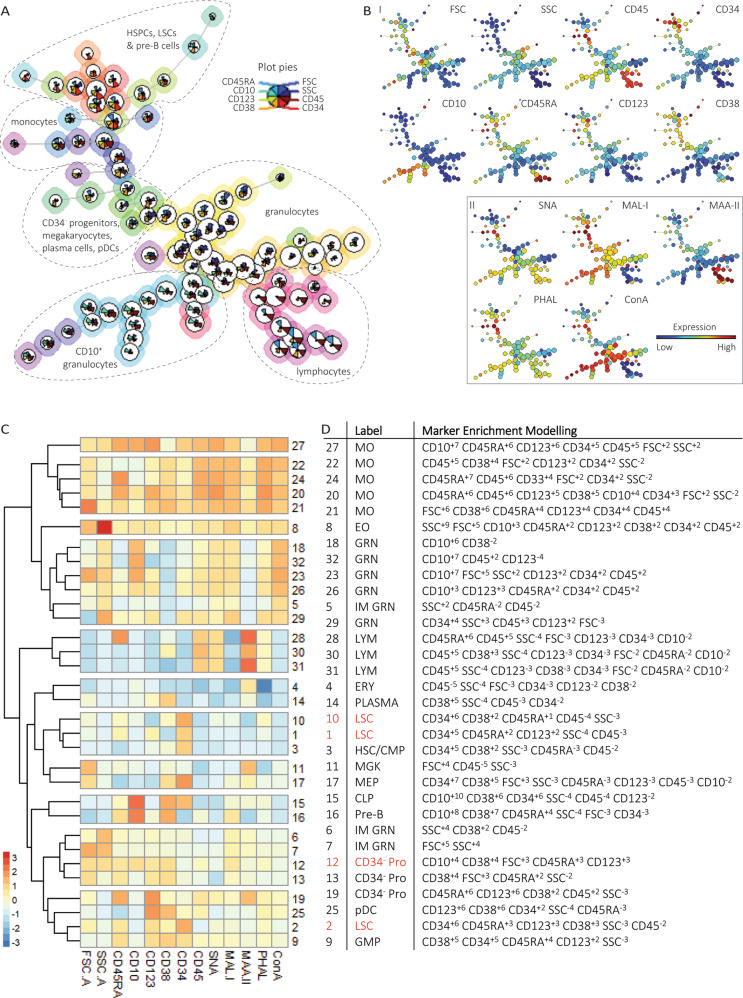
FlowSOM analysis of bone marrow leukocytes with projection of lectin binding intensities reveals glycosylation patterns associated with cell maturation and lineage differentiation. **A** The algorithm FlowSOM clustered the aggregated dataset containing 40 ∙ 10^6^ cells from NBM and patients with MDS, AML and iron deficiency and dysregulated iron metabolism (IDef) into 32 populations based on the backbone markers (Table S2). The background color of the cell clusters (*n* = 90) indicates their population (*n* = 32). The height of the plot pie visualizes the expression of the surface markers and the scatter properties. For visualization purpose, subsets of the minimal spanning tree are encircled (dotted line) with manual labels. **B** FlowSOM minimal spanning tree colored by the scatter intensities, antigen expressions and lectin binding intensities as a marker for glycan expressions for healthy controls. I. The scatter intensities and antigen expressions were used as input for cell clustering. II. The lectin binding intensities were projected on the FlowSOM minimal spanning tree. **C** Heatmap summary of protein expressions, scatter properties and lectin binding intensities for each of the 32 populations derived from all samples. **D** Table summary of the 32 populations as identified by FlowSOM. The populations were manually assigned to cell subsets based on biaxial dotplots, immunophenotypic criteria and the metric MEM. MO monocytes, pDC plasmacytoid dendritic cell, MEP megakaryocyte erythroid progenitor, HSC/CMP hematopoietic stem cell/common myeloid progenitor, LSC leukemic stem cell, GMP granulocyte macrophage progenitor, ERY erythrocyte, MGK megakaryocyte, PC plasma cell, LYM lymphocyte, CLP common lymphoid progenitor, GRN granulocyte, IM immature, EO eosinophil, PRO progenitor, MEM marker enrichment modeling.

The algorithm FlowSOM identified 90 clusters stratified into 32 populations based on scatters and antigen expressions (Fig. 1A–C) [7]. The populations are further referred to by their number in square brackets. We labeled populations using biaxial dotplots and the metric Marker Enrichment Modeling (Fig. 1D) [8]. Comparing frequencies across diagnosis, we identified four populations predominantly found in MDS and AML (further referred to as aberrant) and 28 populations also present in NBM (Fig. S2, Table S3). Latter populations included HSCs together with common myeloid progenitors (HSCs/CMPs), granulocyte-macrophage progenitors (GMPs), megakaryocyte-erythroid progenitors (MEPs), common lymphoid progenitors (CLPs), CD34-negative progenitors, pre-B cells and mature subsets. Aberrant populations included leukemic stem cells (LSCs) and CD34^-^ progenitors with a mixed myeloid/lymphoid phenotype. Compared to NBM, MDS had decreased percentages of CLPs [15], pre-B cells [16] and granulocytes [5,18,29], but increased percentages of HSCs/CMPs [3], LSCs [1], CD34^-^ progenitors [13], lymphocytes [30], plasma cells [14] and monocytes [21]. Patients with AML had increased percentages of HSCs/CMPs [3], LSCs [1,2,10], GMPs [9] and CD34^-^ progenitors [12,19] at the expense of granulocytes [5–7,18,29,32]. Aforementioned perturbations were expected from literature.

## NBM

We projected lectin-binding intensities on the identified populations (Fig. 1B). Questioning whether glycoconjugates distinguish between hematopoietic subsets, we selected NBMs and compared glycan expression on clusters (*n* = 90) between populations (Table S4). Compared to other populations, HSCs/CMPs [3] modestly expressed PHA-L-bound tetra-antennary *N*-glycans, MAL-I-bound α2-3 *N*-linked sialoglycans and MAA-II-bound α2-3 *O*-linked sialoglycans besides lowered amounts of SNA-bound α2-6 sialoglycans and ConA-bound high-mannose glycans and/or di-antennary *N*-glycans. While differentiation to CLPs [15] and MEPs [17] was not accompanied by altered glycosignatures, GMPs [9] showed increased tetra-antennary *N*-glycans, α2-6 sialoglycans and α2-3 *N*-linked sialoglycans (Fig. S3). Immature granulocytes [5,6] showed modest expression of high-mannose glycans and/or di-antennary *N*-glycans, whereas CD10^+^ granulocytes [18,23] were more heavily glycosylated and sialylated. Monocytes [20–22] showed the highest expression of α2-6 and α2-3 *N*-linked sialoglycans and tetra-antennary *N*-glycans. Unlike myeloid populations, lymphocytes [28,31] were characterized by α2-3 *O*-linked sialyation. In brief, the NBM glycosignature showed increasing expression of tested glycan epitopes upon hematopoietic differentiation and maturation, ranging from modest glycosylation of HSPCs to enhanced α2-3 *O*-linked sialyation on lymphocytes and high mannose glycosylation and/or di-antennary *N*-glycosylation on monocytes and granulocytes.

## AML and MDS

**Fig. 2:**
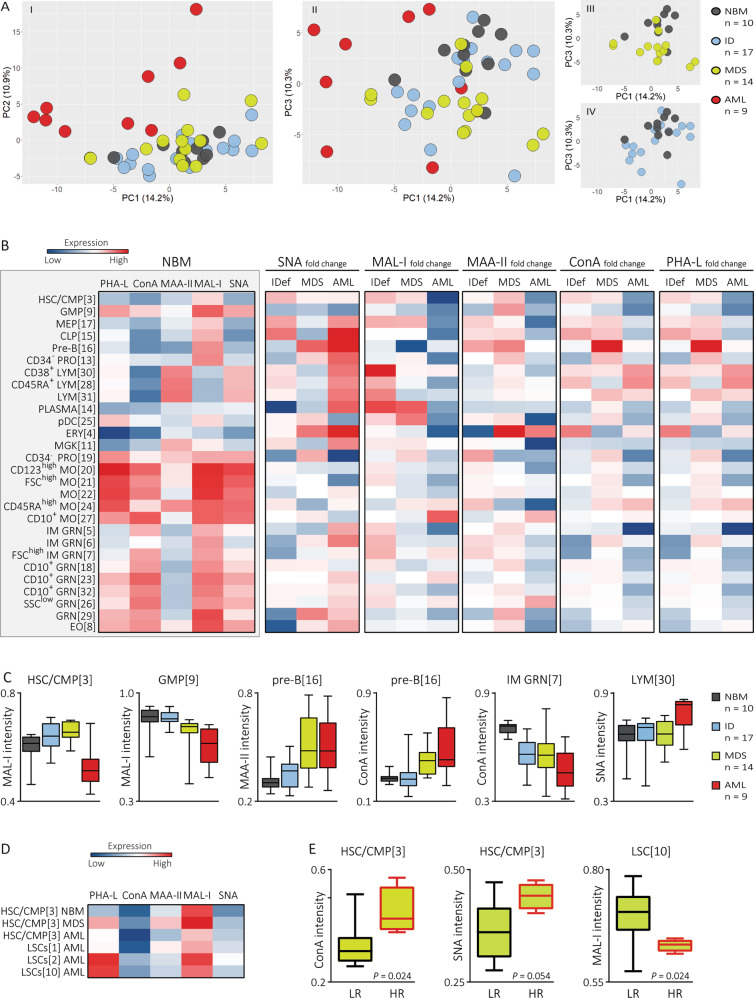
MDS and AML bone marrow leukocytes demonstrate aberrant glycosylation patterns. **A** Principal component (PC) analysis on the lectin binding intensities for each sample indicates dissimilar glycosylation patterns between NBM and patients with AML, MDS and, to a lesser extent, iron deficiency and dysregulated iron metabolism (IDef). Missing values resulted from the absence of a cluster of cells in some of the samples and were dealt with by zero imputation. The first, second and third PC account for 14.2%, 10.9% and 10.3% of the variance, respectively. (I) The second PC separates AML patients from NBM as well as IDef and MDS patients. (II) MDS patients are placed in a distinct region than AML patients and NBM. (III-IV) The third PC separates the majority of the MDS patients and about the half of the IDef patients from NBM. **B** Heatmap summary of the lectin binding intensities on normal populations, i.e. excluding LSCs [1,2,10] and aberrant CD34^-^ progenitors [12]. The first heatmap (gray background) summarizes the lectin binding intensities on NBM-derived populations. The other heatmaps present the difference in lectin-binding intensities in patients samples expressed by the fold change defined as (Y-X)/X using the lectin binding in NBM and patient diagnoses as X and Y, respectively. **C** Boxplots illustrating the median and range of glycan expression on distinct hematopoietic populations across diagnoses. Note that only a selection of the differentially expressed glycans is shown as an example. **D** Heatmap summary presenting lectin binding intensities from NBM-derived HSC/CMPs [3] as compared to aberrant stem cell populations, including AML-derived LSCs [1,2,10] and IDef-, MDS- and AML-derived HSC/CMPs [3]. **E** Boxplot summary of differentially expressed SNA-bound α2-6 sialoglycans, ConA-bound high-mannose glycans and/or di-antennary *N*-glycans and MAL-I-bound α2-3 *N*-linked sialoglycans between low risk (LR, good risk cytogenetics) and high risk (HR, intermediate or poor risk cytogenetics) MDS. The *P* values are based on the Mann–Whitney U test. MO monocytes, pDC plasmacytoid dendritic cell, MEP megakaryocyte erythroid progenitor, HSC/CMP hematopoietic stem cell/common myeloid progenitor, LSC leukemic stem cell, GMP granulocyte macrophage progenitor, ERY erythrocyte, MGK megakaryocyte, PLASMA plasma cell, LYM lymphocyte, CLP common lymphoid progenitor, GRN granulocyte, IM immature, EO eosinophil, PRO progenitor.

To explore glycosylation in myeloid disorders, we applied a principal component analysis on the lectin-binding intensities of the populations for each sample (Fig. 2A). This analysis discriminated AML from other samples, indicating that AML-BM is characterized by a unique glycosignature. Whereas IDef showed overlap with NBM and MDS, MDS partly grouped together in-between NBM and AML. In search of aberrant glycoprofiles that separate MDS from other samples, we compared lectin-binding intensities on populations between diagnoses (Table S5A–C). This revealed aberrant glycan expression on hematopoietic cells across distinct maturational stages and cell lineages in MDS and AML (Fig. 2B). A common feature of MDS and AML was decreased α2-3 *N*-linked and α2-6 sialylation of GMPs [9] and aberrant expression of high-mannose glycans and/or di-antennary *N*-glycans, with increased expression on lymphoid populations [16,30,31] and reduced expression on myeloid subsets, particularly immature granulocytes [5–7] (Fig. 2C).

## AML

Decreased α2-3 *N*-linked sialylation distinguished AML-derived HSCs/CMPs [3], GMPs [9], CLPs [15], pre-B cells [16], CD34^-^ progenitors [13] and mature myeloid populations from NBM (Table S5A). Also LSCs [1] demonstrated decreased α2-3 *N*-linked sialyation, while LSCs [2,10] showed upregulated tetra-antennary *N*-glycosylation (Fig. 2D). Furthermore, AML-derived mature populations showed aberrant tetra-antennary *N*-glycosylation, including increased expression on granulocytes[6,26] and reduced expression on monocytes [20–22]. Lymphoid populations [15,16,28,30,31] from AML patients showed increased α2-6 sialylation.

## MDS

Compared to NBM and AML, MDS-derived HSCs/CMPs [3] were heavily decorated with α2-3 *N*-linked sialic acids and tetra-antennary *N*-glycans (Table S5B). Upregulated tetra-antennary *N*-glycosylation appeared to be propagated to MEPs [17], CLPs [15], pre-B cells [16], lymphocytes [28,30,31] and granulocytes [5–7,18,23,32]. Reduced pre-B cells and dysplastic neutrophils are hallmarks of MDS. Interestingly, MDS-derived pre-B cells showed decreased α2-3 *N*-linked sialyation and enhanced α2-3 *O*-linked sialyation, tetra-antennary *N*-glycosylation, and high-mannose glycosylation and/or di-antennary *N*-glycosylation. Beside upregulated tetra-antennary *N*-glycosylation, MDS-derived immature granulocytes[5–7] showed reduced expression of α2-6 sialic acids and high-mannose glycans and/or di-antennary *N*-glycans. Questioning whether aberrant glycosylation could characterize unfavorable MDS, we compared HSPC glycosignatures between MDS patients classified as low-risk (*n* = 4) and high-risk (*n* = 10) based on cytogenetics. Interestingly, high-risk MDS showed downregulated α2-3 *N*-linked sialyation on LSCs[10] and upregulated α2-6 sialic acids and high-mannose glycans and/or di-antennary *N*-glycans on HSCs/CMPs [3] (Fig. 2E).

## IDef

Glycosignatures from IDef largely resembled patterns found in MDS, although less pronounced (Table S5C). Amongst others, abnormalities included overexpressed tetra-antennary *N*-glycans on lymphocytes [28,30] and reduced expression of high-mannose glycans and/or di-antennary *N*-glycans on granulocytes [5–7,29]. Unlike MDS and AML, IDef had no significantly different glycosignature at HSCs.

This study used unsupervised clustering to explore glycoprofiles in MDS and AML (Fig. S4). Conform a previous study on cord blood, we identified cell type- and maturational stage-specific glycosignatures in NBM [9]. Furthermore, we revealed that altered glycosylation is already detectable at HSCs in AML and MDS. Whereas α2-3 *N*-linked sialylation was increased on MDS-derived HSCs, it was reduced on HSCs in AML. The α2-3 *N*-linked sialic acids have been described to impair CD44-mediated binding to the extracellular matrix, thereby supporting HSPC migration and potentially hampering hematopoietic differentiation [10]. Upregulated α2-3 sialyation in breast cancer affected cell migration by supporting metastatic spread [11]. Contrarily, downregulated α2-3 sialyation was demonstrated in colorectal cancer, indicating that altered glycan expression depends on the tumor type [12]. In line with this hypothesis, a study on leukemic cell lines showed enhanced α2-3 sialylation within erythroid leukemia (M6) and reduced expression in myeloid (PLB985) and promyelocytic leukemia (HL60) [5]. We observed increased tetra-antennary *N*-glycosylation on most hematopoietic populations in MDS and on CLPs in AML. Tetra-antennary *N*-glycosylation has been linked to the suppressive potency of regulatory T-cells [13]. This suggests that increased tetra-antennary *N*-glycosylation on lymphoid subsets may play a role in tumor surveillance in MDS and AML, whereas general overexpression throughout hematopoiesis may be related to myelodysplastic phenotypes. Compared to low-risk, high-risk MDS showed upregulated α2-6 sialyation at HSC level. Differently, we observed increased α2-6 sialyation on AML-derived lymphoid cells. Previous literature showed that overexpression of *STGAL1*, the gene encoding for α2,6-sialyltransferase, facilitates progression of prostate cancer [14]. Another study showed higher α2-6 sialyation on tolerogenic DCs that is downregulated after DC maturation with proinflammatory cytokines [15]. We hypothesize that enhanced α2-6 sialylation on lymphoid cells may induce tumor surveillance.

To conclude, this study suggests that increased tetra-antennary *N*-glycosylation contributes to myelodysplastic phenotypes, whereas decreased α2-3 *N*-linked sialyation and increased α2-6 sialyation characterizes AML. Upregulation of α2-6 sialic acids and high-mannose glycans and/or di-antennary N-glycans on HSCs differed high-risk from low-risk MDS, indicating that glycoprofiles could be of value for MDS risk stratification. However, the main disadvantage of this exploratory study is the low sample size and power. Larger studies that combine profiling of antigen and lectin intensities with mass spectrometry and sequencing of glycosyltransferases are warranted.

## Supplementary information

Supplemental File

## Data Availability

The data that support the findings of this study are available from the corresponding author, A. A. van de Loosdrecht, on reasonable request.
