# Supplementary material for: Dysregulation of developmental and cell type-specific expression of glycoconjugates on hematopoietic cells: a new characteristic of myelodysplastic neoplasms (MDS)
Source: Leukemia. 2023 Feb 9;37(3):702–7. doi: 10.1038/s41375-022-01784-x (PMC9991906; doi:10.1038/s41375-022-01784-x)
Supplement: Supplementary file 1 — Supplemental File [file 41375_2022_1784_MOESM1_ESM.pdf]

Dysregulation of developmental and cell type-specific expression of glycoconjugates on hematopoietic cells: a new characteristic of myelodysplastic neoplasms (MDS)

Table of Contents

Sample selection	page 3
Flow cytometry	page 3
Data preprocessing	page 3
Clustering analysis	page 4
Statistical analysis	page 4
Table S1. Clinical characteristics of MDS patients	page 5
Table S2. Flow cytometry panel	page 6
Table S3. Differences in cell population frequencies across diagnosis	page 7
Table S4. Statistical difference in lectin-binding intensities on populations in NBM	page 8
Table S5A. Statistical difference in lectin-binding intensities between NBM and AML	page 9
Table S5B. Statistical difference in lectin-binding intensities between NBM and MDS	page 10
Table S5C. Statistical difference in lectin-binding intensities between NBM and IDef	page 11
Figure S1. Methodological summary from data acquisition to cell clustering	page 12
Figure S2. Population frequencies stratified by diagnosis	page 13
Figure S3. Lectin intensities on NBM-derived hematopoietic populations	page 14
Figure S4. Graphical summary	page 15

Sample selection

In total, 58 bone marrow (BM) samples from patients suspected of MDS and AML in addition to 11 normal bone marrows (NBMs) were collected between March, 2016 and April, 2019. After diagnostic work-up, MDS and AML was confirmed in 15/58 and 9/58 patients, respectively. Cytopenia due to iron deficiency or dysregulated iron metabolism (IDef) was diagnosed in 20/58 patients. Patients with inconclusive diagnoses (6/58), lymphoid neoplasms (6/58) and myeloproliferative neoplasms (2/58) were excluded. From the selected MDS, AML and IDef patients and NBMs, another 1 NBM, 1 MDS and 3 IDef samples were excluded because of a suboptimal titration of the lectin stainings in the first experiments. Accordingly, the study population comprised 14 MDS patients (Table S1), 9 AML patients, 17 IDef patients and 10 NBMs.

Sample preparation

Fresh BM samples were collected in heparin tubes. For each sample, $10 \cdot 10^6$ white blood cells (WBCs) were deprived of erythrocytes using an ammonium chloride solution (PharmLyse; BD Biosciences) for 10 minutes at room temperature. Lysed samples were washed twice and resuspended in TSM buffer (20mM Tris-HCl (pH 7.4), 150mM NaCl, 1mM CaCl2) with 0.1% human serum albumin (HSA).

Flow cytometry

Used fluorescent-conjugated monoclonal antibodies and lectins are summarized in Table S2. Prepared cell suspensions were divided over 6 tubes, incubated with biotin-labelled lectins for 60 minutes at 4°C in the dark and washed twice with TSM buffer with 0.1% HSA. Secondly, samples were incubated with streptavidin-AF647 and the antibody backbone for 30 minutes at 4°C, washed twice and resuspended in TSM buffer with 0.1% HSA. Data acquisition was performed using a BD FACSCanto II flow cytometer (BD Biosciences, San Jose, CA) equipped with 3 lasers (405nm, 488nm and 633nm).

Data preprocessing

The methodological procedure is presented in Figure S1. Flow cytometry data were manually pregated on $CD45^+$ leukocytes using Infinicyt software 1.8 (Cytognos, Salamanca, Spain). Pregated fcs files were subjected to preprocessing steps to detect technical errors potentially hindering biological interpretation. This quality control was performed using the R package FlowAI that accounts for amongst others abrupt fluctuations in the flow rate, signal acquisition instability, and outliers and margin events. Protein marker expression data and lectin binding intensities were compensated using the spillover matrix from the fcs file and transformed using hyperbolic arcsin with a cofactor of 150. Range scaling (0.05-99.5%) between files

was performed to decrease sensitivity with regard to outliers. The median cell count of all files was $1.6{\cdot}10^5$ pre-gated WBCs per file. Preprocessed files were randomly subsampled to a maximum of $1.6{\cdot}10^5$ cells and aggregated. The complete dataset contained $40{\cdot}10^6$ cells from 50 subjects. No clustering was observed with respect to time of data acquisition.

Clustering analysis

The unsupervised tool FlowSOM was used to cluster the dataset into 32 cell populations based on scatter intensities and antigen expressions.[1] The lectin binding intensities were not used as input for cell clustering, but projected on the FlowSOM minimal spanning tree. The FlowSOM version 1.18.0 was used in R. A general demonstration of the R code of the FlowSOM analysis pipeline is available on Github.

Statistical analysis

The frequencies of the 32 populations and expressions of the 5 lectins on the separate populations were statistically compared between NBMs and patients. The dataset contained 192 continuous variables that were both normally and non-normally distributed. The Mann-Whitney U test was applied for comparisons between two groups to search for glycosylation patterns characteristic for diagnostic cohorts. Two-sided *P*-values <0.050 were considered statistically significant. Analyses were conducted with the Statistical Package for the Social Sciences version 22.

Table S1. Clinical characteristics of MDS patients

UPN	Sex	Age *years*	WHO	IPSS-R	CCSS	Hb *g/dL*	ANC *·10^9/L*	WBC *·10^9/L*	PLT *·10^9/L*	BM-BL *%*	PB-BL *%*
MDS02	F	41	RS-MLD	Low	Good	9.0	1.7	4.8	180	3	0
MDS03	M	78	RS-MLD	Int	Inter	9.7	4.9	6.5	>100	4	0
MDS04	F	78	MLD	≥ Int	Good	4.2	4.4	7.8		3	0
MDS05	M	63	EB-2	Very high	Good	7.4	0.1	3.3	24	14	0
MDS06	F	48	RS-MLD	Low	Good	6.6	2.9	6.5	422	0	0
MDS07	M	62	EB-1	NA	Good					5 [1]	0
MDS08	M	73	MLD	Low	Good	7.3	3.4	3.8	243	2	0
MDS09	M	63	RS-MLD	Low	Good	8.4	2.6	5,2	21	2	0
MDS10	M	88	SLD	Very low	Good	10.1	5.0	7.3	123	1	0
MDS11	M	66	MLD	Unknown	Good					1	0
MDS12	M	69	MLD	Inter	Inter	6.6	62.7	93.6	90	1	0
MDS13	F	61	RS-MLD	Low	Good	5.8	3.1	5.2	517	1	0
MDS14	M	76	RS-SLD	Int	Poor	10.0	4.7	7.5	198	1	0
MDS15	M	80	MDS-U	Int	Inter	9.7	1,7	6.1	46	1	1

[1] Bone marrow myeloblast percentage assessed by flow cytometry. Abbreviations: WHO, World Health Organization; IPSS-R, revised International Prognostics Scoring System; CCSS, New Comprehensive Cytogenetic Scoring System; Hb, hemoglobin; ANC, absolute neutrophil count; WBC, white blood cells; PLT, platelets; BM-BL, bone marrow blasts; PB-BL, peripheral blood blasts; SLD, single lineage dysplasia; MLD, multilineage dysplasia; RS-SLD, single lineage dysplasia with ring sideroblasts; RS-MLD, multilineage dysplasia with ring sideroblasts; EB-1, excess blasts type 1; EB-2, excess blasts type 2; MDS-U, MDS, unclassifiable; Int, intermediate; NA, not applicable

Table S2. Flow cytometry panel

	APC-H7	HV500c	BV421	PC7	PerCP Cy5.5	FITC	PE	AF647	Glycans
1	L/D	CD45	CD34	CD38	CD123	CD45RA	CD10		
2	L/D	CD45	CD34	CD38	CD123	CD45RA	CD10	PHA-L	tetra-antennary *N*-glycans
3	L/D	CD45	CD34	CD38	CD123	CD45RA	CD10	ConA	high-mannose glycans and di-antennary N-glycans
4	L/D	CD45	CD34	CD38	CD123	CD45RA	CD10	MAA-II	α2-3 *O*-linked sialic acids
5	L/D	CD45	CD34	CD38	CD123	CD45RA	CD10	MAL-I	α2-3 *N*-linked sialic acids
6	L/D	CD45	CD34	CD38	CD123	CD45RA	CD10	SNA	α2-6 sialic acids

The flow cytometry panel consisted of six tubes. The first tube was a fluorescence minus one (FMO) control containing the antibody backbone, but no lectin. The other tubes contained various lectins in addition to the backbone markers. The lectins PHA-L, ConA and SNA recognize tetra-antennary *N*-glycans, high-mannose glycans and di-antennary *N*-glycans, and α2-6 sialoglycans, respectively. The MAA-II and MAL-I lectins bind to α2-3 sialoglycans with distinct carbohydrate binding specificities: MAA-II has a preference for *O*-linked α2-3 sialic acids and MAL-I for *N*-linked α2-3 sialic acids.[2] The biotinylated lectins were purchased from Vector Laboratories (PHA-L, cat. nr. B-1115; ConA, cat. nr. B-1005; MAA-II, cat. nr. B-1265; MAL-I, cat. nr. B-1315; SNA, cat. nr. B-1305). The following antibodies were applied: CD45, clone 2D1 cat.nr. 655873; CD34, clone 581 cat. nr. 562577 (all BD Biosciences); CD38, clone LS198-4-3 cat. nr. B49198 (Beckman Coulter) CD123, clone 7G3 cat. nr. 558714; CD45RA, clone L48 cat. nr. 335039; CD10, clone SS2/36, cat. nr. R084801 (DAKO). The life/death marker (Fixable Viable Stain 780) was obtained from R&D Systems. Abbreviations: L/D, life/death marker; PHA-L, Phytohemagglutinin-L; ConA, Concanavalin A; MAA-II, *Maackia amurensis* agglutinin II; MAL-I, *Maackia amurensis* leucoagglutinin I; SNA, *Sambucus nigra* agglutinin.

Table S3. Differences in cell population frequencies across diagnosis

Normal populations		NBM vs. IDef	NBM vs. MDS	NBM vs. AML	MDS vs. IDef	MDS vs. AML
3	HSCs/CMPs	ns	0.036 ↑	0.028 ↑	0.015 ↑	ns
4	Erythrocytes	0.023 ↑	<0.001 ↑	0.065 ↑	ns	ns
5	CD10^{-} Granulocytes	ns	0.048 ↓	<0.001 ↓	ns	<0.001 ↓
6	CD10^{-} Granulocytes	ns	ns	<0.001 ↓	ns	<0.001 ↓
7	FSC^{high} CD10^{-} Granulocytes	ns	ns	0.017 ↓	ns	0.002 ↓
8	Eosinophils	ns	ns	0.043 ↑	ns	0.062 ↑
9	GMPs	ns	ns	<0.001 ↑	ns	0.002 ↑
11	Megakaryocytes	ns	0.084 ↑	ns	ns	0.062 ↓
13	CD34^{-} Progenitors	0.020 ↑	0.026 ↑	ns	ns	ns
14	Plasma cells	ns	0.036 ↑	ns	0.048 ↑	ns
15	CLPs	ns	0.003 ↓	0.079 ↓	0.017 ↓	ns
16	Pre-B cells	ns	0.009 ↓	0.017 ↓	0.010 ↓	ns
17	MEPs	ns	ns	0.079 ↓	ns	ns
18	CD10^{+} Granulocytes	ns	0.042 ↓	<0.001 ↓	0.019 ↓	0.001 ↓
19	CD34^{-} Progenitors	ns	ns	0.003 ↑	ns	0.009 ↑
20	CD123^{high} Monocytes	ns	ns	ns	ns	ns
21	FSC^{high} Monocytes	0.052 ↑	0.016 ↑	0.028 ↑	ns	ns
22	Monocytes	ns	ns	0.043 ↓	ns	0.033 ↓
23	CD10^{+} Granulocytes	ns	ns	ns	ns	0.028 ↓
24	CD45RA^{high} Monocytes	ns	ns	0.017 ↓	ns	ns
25	pDCs	ns	ns	ns	ns	ns
26	SSC^{low} CD10^{+} Granulocytes	<0.001 ↑	ns	0.079 ↓	0.064 ↓	0.028 ↓
27	CD10^{+} Monocytes	0.083 ↑	ns	ns	ns	ns
28	CD45RA^{+} Lymphocytes	ns	ns	ns	ns	0.062 ↓
29	Granulocytes	ns	0.042 ↓	<0.001 ↓	ns	0.002 ↓
30	CD38^{+} Lymphocytes	ns	0.007 ↑	ns	0.008 ↑	0.023 ↓
31	Lymphocytes	ns	ns	0.065 ↓	ns	0.053 ↓
32	CD10^{+} Granulocytes	ns	ns	<0.001 ↓	ns	0.003 ↓
**Abnormal populations**		**NBM vs. IDef**	**NBM vs. MDS**	**NBM vs. AML**	**MDS vs. IDef**	**MDS vs. AML**
1	LSCs	0.001 ↑	<0.001 ↑	<0.001 ↑	ns	<0.001 ↑
2	LSCs	0.052 ↑	0.096 ↑	<0.001 ↑	ns	0.001 ↑
10	LSCs	ns	ns	<0.001 ↑	ns	<0.001 ↑
12	CD34^{-} Aberrant progenitors	0.083 ↑	ns	0.001 ↑	ns	0.023 ↑

The population frequencies are determined from the second tube of the flow cytometry panel and relative to the CD45^{+} leukocytes compartment. Populations are classified as abnormal or normal based on their frequency in patients as compared to NBM. *P*-values are presented for significant values (*P*<0.050) and for trends towards significant values (*P*=0.050-0.100), whereas *P*-values above 0.100 are presented as ns (not significant). Arrows indicate increased or decreased population frequencies.

Table S4. Statistical difference in lectin-binding intensities on cell clusters between populations in NBM

Normal populations		PHA-L	ConA	MAA-II	MAL-I	SNA
3	HSCs/CMPs	ns	0.084 ↓	ns	ns	0.024 ↓
4	Erythrocytes	0.044 ↓	ns	ns	ns	0.022 ↓
5	CD10$^-$ Granulocytes	<0.001 ↓	0.063 ↑	<0.001 ↓	0.051 ↓	ns
6	CD10$^-$ Granulocytes	0.043 ↓	ns	ns	ns	0.023 ↓
8	Eosinophils	ns	0.089 ↑	ns	ns	ns
11	Megakaryocytes	0.036 ↓	ns	0.060 ↑	ns	ns
15	CLP	ns	ns	ns	ns	0.089 ↓
18	Granulocytes	ns	0.006 ↑	<0.001 ↓	0.090 ↑	ns
20	Monocytes	0.022 ↑	0.067 ↑	ns	0.022 ↑	0.022 ↑
21	Monocytes	0.067 ↑	ns	ns	0.067 ↑	0.044 ↑
22	Monocytes	<0.001 ↑	0.085 ↑	ns	0.001 ↑	0.047 ↑
23	Granulocytes	ns	0.003 ↑	ns	0.098 ↑	0.045 ↑
24	Monocytes	0.089 ↑	ns	ns	ns	0.089 ↑
26	Granulocytes	ns	0.040 ↑	ns	ns	ns
27	Monocytes	ns	0.044 ↑	ns	ns	ns
28	CD45RA$^+$ Lymphocytes	ns	<0.001 ↓	<0.001 ↑	0.002 ↓	0.020 ↑
29	Granulocytes	0.098 ↑	ns	ns	0.018 ↑	ns
31	Lymphocytes	ns	<0.001 ↓	<0.001 ↑	<0.001 ↓	ns

The expression of PHAL-bound tetra-antennary *N*-glycans, ConA-bound high-mannose glycans and di-antennary *N*-glycans, MAA-II-bound α2-3 O-linked sialoglycans, MAL-I-bound α2-3 *N*-linked sialoglycans and SNA-bound α2-6 sialoglycans on NBM-derived cell clusters (n = 90). Only normal populations (i.e. exclusion of 4 aberrant populations predominately present in patients) and populations with a trend towards statistical significance or with a significant difference are shown. *P*-values are presented for significant values (*P*=<0.050) and for trends towards significant values (*P*=0.050-0.100), whereas *P*-values above 0.100 are presented as ns (not significant). Arrows indicate increased or decreased expression.

Table S5A. Statistical difference in lectin-binding intensities on populations between AML and NBM

	Normal populations	PHAL	ConA	MAA-II	MAL-I	SNA
3	HSCs/CMPs	ns	ns	ns	0.022 ↓	ns
4	Erythrocytes	ns	ns	ns	ns	0.002 ↑
5	CD10$^-$ Granulocytes	ns	0.006 ↓	ns	ns	ns
6	CD10$^-$ Granulocytes	0.053 ↑	0.035 ↓	ns	ns	ns
7	CD10$^-$ Granulocytes	ns	<0.001 ↓	ns	ns	ns
9	GMPs	0.095 ↓	<0.001 ↓	ns	0.001 ↓	0.002 ↓
11	Megakaryocytes	0.008 ↑	0.017 ↓	0.008 ↓	ns	ns
13	CD34$^-$ Progenitors	ns	0.065 ↓	ns	0.004 ↓	ns
14	Plasma cells	ns	0.008 ↓	ns	ns	ns
15	CLPs	0.013 ↑	ns	ns	0.025 ↓	0.070 ↑
16	Pre-B cells	ns	0.004 ↑	0.003 ↑	0.006 ↓	0.008 ↑
17	MEPs	ns	ns	0.095 ↓	ns	ns
19	CD34$^-$ Progenitors	0.017 ↓	0.065 ↓	ns	ns	ns
20	Monocytes	0.003 ↓	ns	ns	ns	ns
21	Monocytes	0.053 ↓	0.035 ↓	ns	0.003 ↓	0.022 ↓
22	Monocytes	0.001 ↓	ns	ns	0.006 ↓	ns
24	Monocytes	ns	ns	0.006 ↓	ns	ns
26	Granulocytes	0.035 ↑	0.043 ↓	ns	ns	ns
27	Monocytes	0.003 ↑	ns	ns	0.043 ↑	ns
28	CD45RA$^+$ Lymphocytes	ns	0.022 ↑	ns	ns	0.004 ↑
29	Granulocytes	ns	0.017 ↓	ns	<0.001 ↓	ns
30	CD38$^+$ Lymphocytes	0.065 ↑	0.010 ↑	0.006 ↓	ns	0.013 ↑
31	Lymphocytes	ns	0.003 ↑	ns	ns	0.004 ↑

The expression of PHAL-bound tetra-antennary *N*-glycans, ConA-bound high-mannose glycans and di-antennary *N*-glycans, MAA-II-bound α2-3 *O*-linked sialoglycans, MAL-I-bound α2-3 *N*-linked sialoglycans and SNA-bound α2-6 sialoglycans between NBMs versus AML patients. Only 23/32 populations are shown, excluding 4 aberrant populations predominately present in patients (populations 1, 2, 10, 12) and 5 populations without significantly different lectin expressions between NBM and AML (populations 8, 18, 23, 25, 32). *P*-values are presented for significant values (*P*=<0.050) and for trends towards significant values (*P*=0.050-0.100), whereas *P*-values above 0.100 are presented as ns (not significant). Arrows indicate increased or decreased expression.

Table S5B. Statistical difference in lectin-binding intensities on populations between MDS and NBM

	Normal populations	PHAL	ConA	MAA-II	MAL-I	SNA
3	HSCs/CMPs	0.009 ↑	ns	0.096 ↑	0.002 ↑	ns
4	Erythrocytes	ns	ns	ns	ns	0.084 ↑
5	CD10^{-} Granulocytes	0.005 ↑	0.005 ↓	0.096 ↑	ns	0.031 ↓
6	CD10^{-} Granulocytes	<0.001 ↑	0.036 ↓	0.042 ↑	ns	0.084 ↓
7	CD10^{-} Granulocytes	<0.001 ↑	<0.001 ↓	ns	0.056 ↑	0.048 ↓
8	Eosinophils	ns	ns	ns	0.013 ↓	ns
9	GMPs	ns	ns	ns	0.005 ↓	0.022 ↓
11	Megakaryocytes	ns	0.084 ↓	ns	ns	ns
13	CD34^{-} Progenitors	ns	ns	0.084 ↑	ns	ns
14	Plasma cells	0.011 ↑	ns	ns	0.056 ↑	0.007 ↓
15	CLPs	0.014 ↑	0.049 ↑	0.049 ↑	ns	ns
16	Pre-B cells	<0.001 ↑	<0.001 ↑	<0.001 ↑	<0.001 ↓	ns
17	MEPs	0.002 ↑	0.026 ↑	0.042 ↑	0.003 ↑	ns
18	Granulocytes	0.022 ↑	ns	ns	ns	ns
22	Monocytes	0.019 ↓	ns	ns	ns	ns
23	Granulocytes	0.056 ↑	ns	ns	ns	ns
25	pDCs	0.026 ↑	ns	0.074 ↑	0.022 ↑	ns
26	Granulocytes	0.011 ↑	ns	ns	ns	ns
28	CD45RA^{+} Lymphocytes	0.042 ↑	ns	ns	ns	ns
29	Granulocytes	ns	ns	ns	<0.001 ↓	ns
30	CD38^{+} Lymphocytes	0.005 ↑	0.019 ↑	ns	ns	ns
31	Lymphocytes	0.011 ↑	0.056 ↑	ns	ns	ns
32	Granulocytes	0.019 ↑	ns	ns	ns	ns

The expression of PHAL-bound tetra-antennary *N*-glycans, ConA-bound high-mannose glycans and di-antennary *N*-glycans, MAA-II-bound α2-3 *O*-linked sialoglycans, MAL-I-bound α2-3 *N*-linked sialoglycans and SNA-bound α2-6 sialoglycans between NBMs versus MDS patients. Only 23/32 populations are shown, excluding 4 aberrant populations predominately present in patients (populations 1, 2, 10, 12) and 5 populations without significantly different lectin expressions between NBM and AML (populations 19, 20, 21, 24, 27). *P*-values are presented for significant values (*P*=<0.050) and for trends towards significant values (*P*=0.050-0.100), whereas *P*-values above 0.100 are presented as ns (not significant). Arrows indicate increased or decreased expression.

Table S5C. Statistical difference in lectin-binding intensities on populations between IDef and NBM

	Normal populations	PHAL	ConA	MAA-II	MAL-I	SNA
3	HSCs/CMPs	0.074 ↑	ns	ns	0.093 ↑	ns
4	Erythrocytes	ns	0.015 ↑	ns	ns	ns
5	Granulocytes	0.083 ↑	0.035 ↓	ns	ns	ns
6	Granulocytes	ns	<0.001 ↓	ns	ns	0.009 ↓
7	Granulocytes	0.093 ↑	<0.001 ↓	ns	0.046 ↑	0.052 ↓
13	$CD34^-$ Progenitors	ns	0.003 ↓	ns	ns	ns
14	Plasma cells	ns	ns	ns	ns	0.093 ↓
15	CLPs	ns	ns	0.020 ↑	ns	ns
17	MEPs	ns	ns	0.031 ↑	ns	ns
21	Monocytes	ns	ns	ns	0.046 ↑	ns
23	Granulocytes	ns	ns	0.031 ↓	ns	ns
24	Monocytes	ns	ns	0.059 ↑	ns	ns
26	Granulocytes	0.052 ↑	ns	ns	ns	ns
28	$CD45RA^+$ Lymphocytes	0.040 ↑	0.093 ↑	ns	ns	ns
29	Granulocytes	0.093 ↑	<0.001 ↓	ns	ns	0.031 ↓
30	$CD38^+$ Lymphocytes	0.035 ↑	ns	0.093 ↓	ns	ns

The expression of PHAL-bound tetra-antennary *N*-glycans, ConA-bound high-mannose glycans and di-antennary *N*-glycans, MAA-II-bound α2-3 *O*-linked sialoglycans, MAL-I-bound α2-3 *N*-linked sialoglycans and SNA-bound α2-6 sialoglycans between NBMs versus IDef patients. Only 16/32 populations are shown, excluding 4 aberrant populations predominately present in patients (populations 1, 2, 10, 12) and 12 populations without significantly different lectin expressions between NBM and IDef (populations 8, 9, 11, 16, 18, 19, 20, 22, 25, 27, 31, 32). *P*-values are presented for significant values (P=<0.050) and for trends towards significant values (P=0.050-0.100), whereas *P*-values above 0.100 are presented as ns (not significant). Arrows indicate increased or decreased expression.

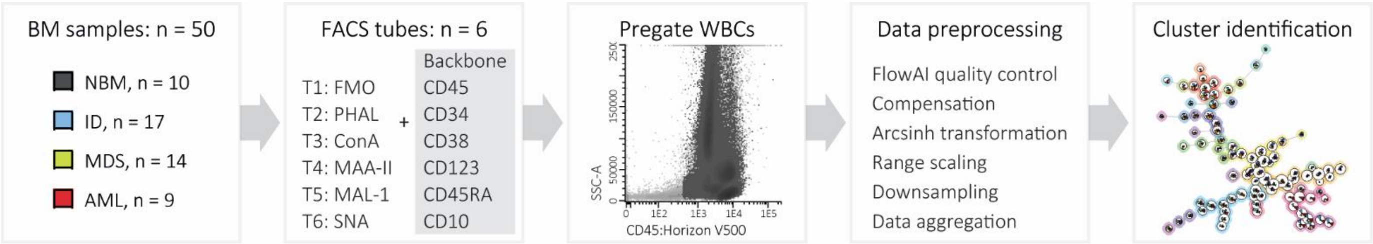

Figure S1. Methodological summary from data acquisition to cell clustering

Of each BM sample, $10{\cdot}10^6$ cells were distributed over 6 tubes including an antibody backbone combined with one lectin or no lectin (FMO). The 300 fcs files (6 files x 50 samples) were manually pre-gated on the WBC compartment that was subsequently subjected to distinct pre-processing steps, including quality control, compensation, arcsinh transformation and range scaling between files. The pre-gated and preprocessed files were downsampled and aggregated to one dataset containing $40{\cdot}10^6$ cells. The unsupervised clustering tool FlowSOM classified the dataset into 32 cell populations based on scatter intensities and antigen expressions. Finally, the lectin binding intensities were projected on the FlowSOM minimal spanning tree. Abbreviations: (N)BM, (normal) bone marrow; IDef, iron deficiency and dysregulated iron metabolism; MDS, myelodysplastic neoplasms; AML, acute myeloid leukemia; FACS, fluorescence-activated cell sorting; PHA-L, Phytohemagglutinin-L; ConA, Concanavalin A; MAA-II, Maackia amurensis agglutinin II; MAL-1, Maackia amurensis leucoagglutinin; SNA, Sambucus nigra agglutinin; WBCs, white blood cells.

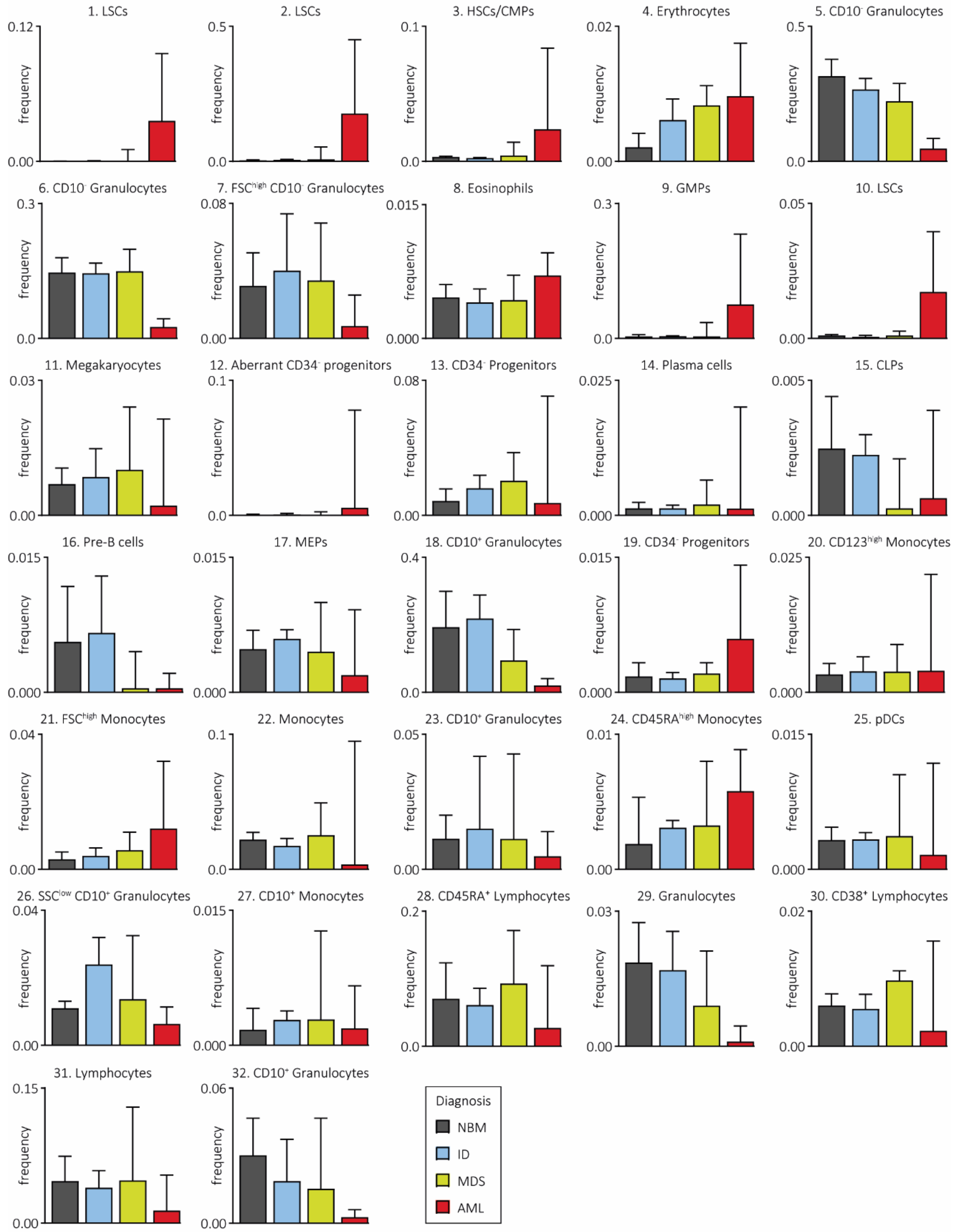

Figure S2. Population frequencies stratified by diagnosis

The population frequencies are determined from the second tube of the flow cytometry panel and expressed relative to the WBC compartment. The relative frequencies are given as fractions between 0 and 1. Except for populations 5 and 6, the populations frequencies followed a non-normal distribution and are presented by the median with the 95% confidence interval. The frequencies of population 5 and 6 followed a normal distribution and are presented as mean with the 95% confidence interval.

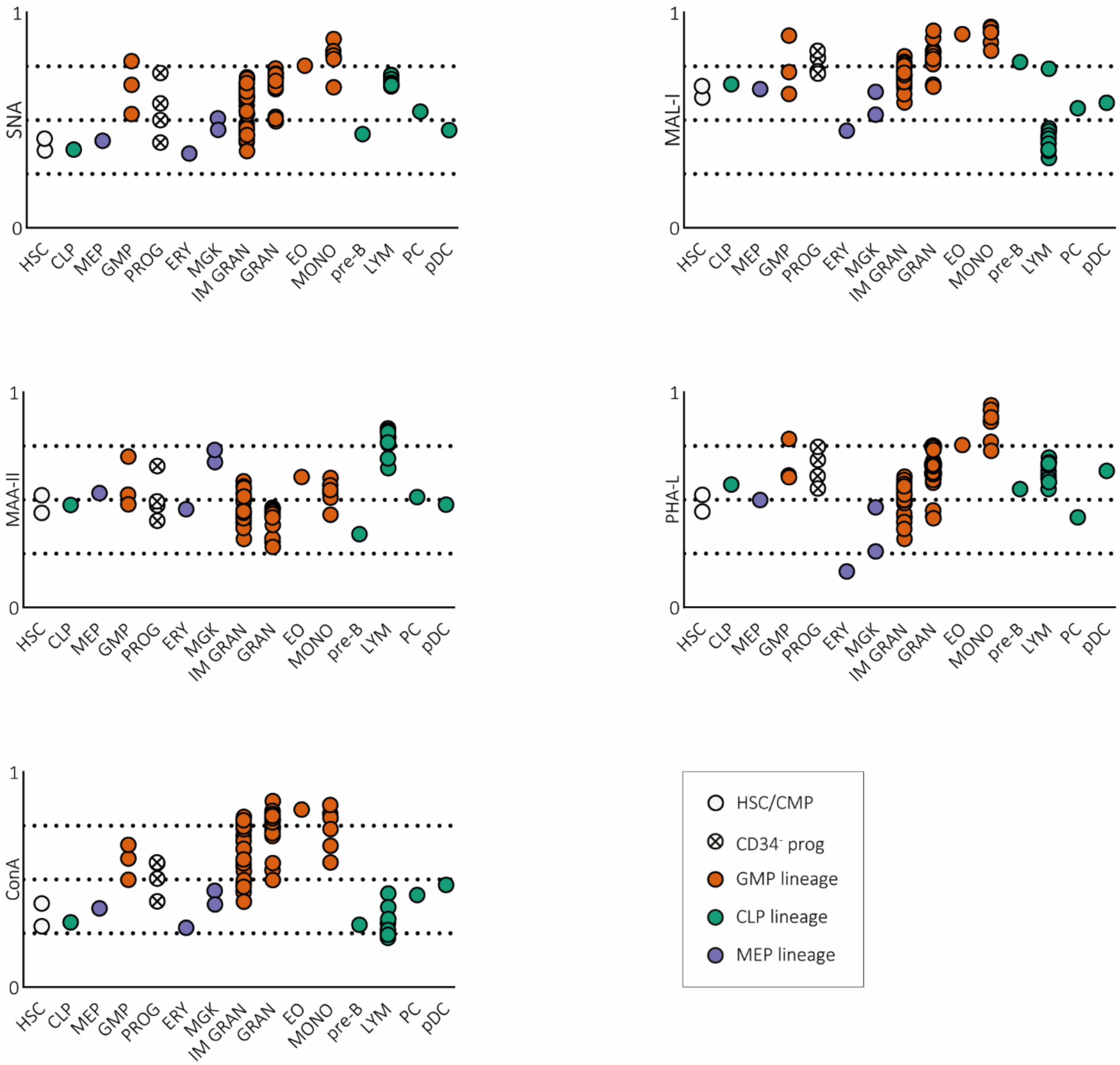

Figure S3. Lectin intensities on NBM-derived hematopoietic populations

The FlowSOM populations were manually grouped in major hematopoietic populations to illustrate lectin binding intensities across hematopoiesis. Only 28/32 populations are shown, excluding 4 aberrant populations predominately present in patients (populations 1, 2, 10, 12) and not in NBMs. The 28 FlowSOM populations are indicated by individual symbols for each manual group. Abbreviations: MONO, monocytes; pDC, plasmacytoid dendritic cell; MEP, megakaryocyte erythroid progenitor; HSC/CMP, hematopoietic stem cell/common myeloid progenitor; GMP, granulocyte macrophage progenitor; ERY, erythrocyte; MGK, megakaryocyte; PC, plasma cell; LYM, lymphocyte; CLP, common lymphoid progenitor; GRN, granulocyte; IM, immature ($CD10^-$); EO, eosinophil; PRO, progenitor

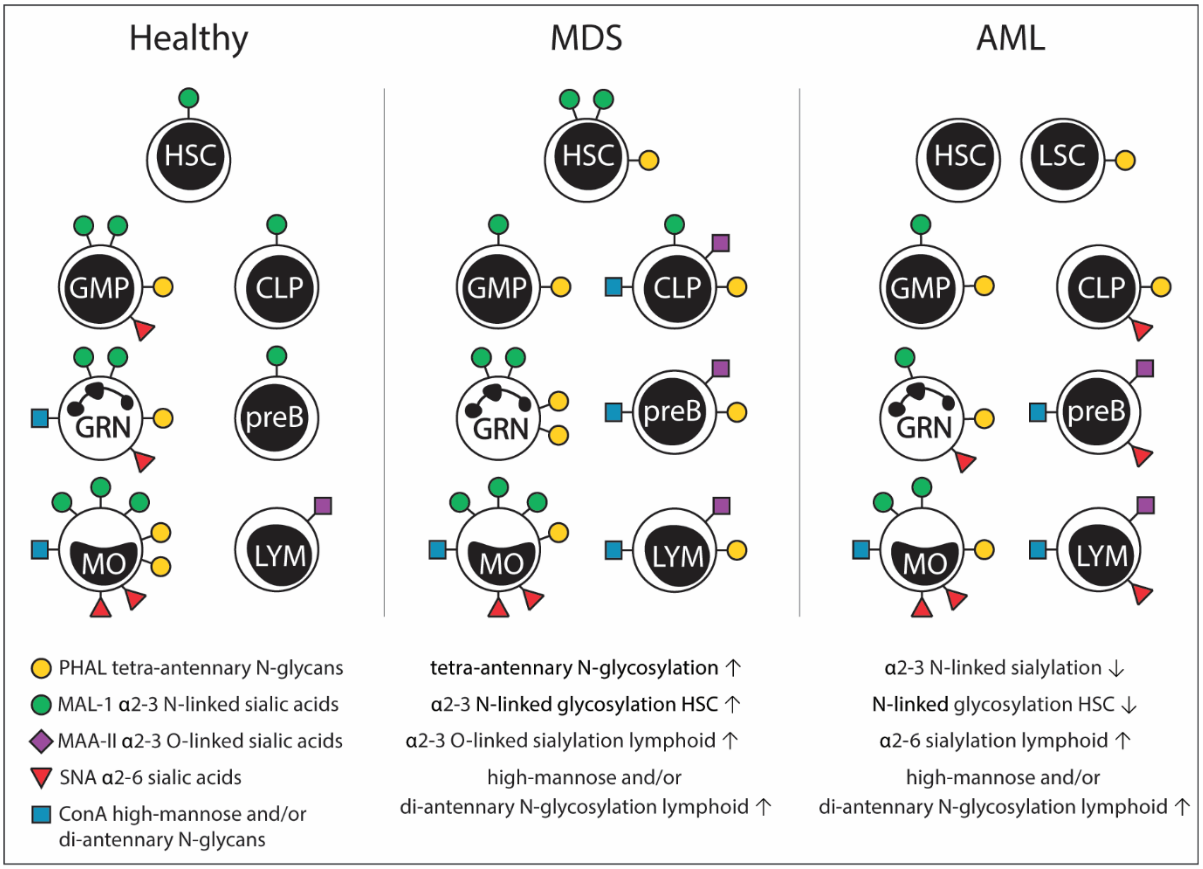

Figure S4. Graphical summary

Dysregulation of developmental and cell type-specific expression of glycoconjugates in MDS. In MDS patients, MAL-I-bound α2-3 *N*-linked sialyation and PHAL-bound tetra-antennary *N*-glycosylation was increased throughout hematopoiesis. In AML patients, MAL-I-bound α2-3 *N*-linked sialoglycans was reduced on immature hematopoietic subsets while SNA-bound α2-6 sialyation was increased on lymphoid cells. Abbreviations: HSC, hematopoietic stem cell; GMP, granulocyte-macrophage progenitor; CLP, common lymphoid progenitor; GRN, granulocyte; preB, pre-B cell; MO, monocyte; LYM, lymphocyte

References

1. Van Gassen S, Callebaut B, Van Helden MJ, Lambrecht BN, Demeester P, Dhaene T*, et al*. FlowSOM: Using self-organizing maps for visualization and interpretation of cytometry data. *Cytometry A* 2015; **87**: 636-45.

2. Geisler C, Jarvis DL. Effective glycoanalysis with Maackia amurensis lectins requires a clear understanding of their binding specificities. *Glycobiology* 2011; **21**: 988-93.
